# Genetic dissection of GABAergic neural circuits in mouse neocortex

**DOI:** 10.3389/fncel.2014.00008

**Published:** 2014-01-27

**Authors:** Hiroki Taniguchi

**Affiliations:** Development and Function of Inhibitory Neural Circuits, Max Planck Florida Institute for Neuroscience, JupiterFL, USA

**Keywords:** GABA, inhibitory interneurons, cortical circuit, gene targeting, Cre drivers, genetic fate mapping, chandelier cell

## Abstract

Diverse and flexible cortical functions rely on the ability of neural circuits to perform multiple types of neuronal computations. GABAergic inhibitory interneurons significantly contribute to this task by regulating the balance of activity, synaptic integration, spiking, synchrony, and oscillation in a neural ensemble. GABAergic interneurons display a high degree of cellular diversity in morphology, physiology, connectivity, and gene expression. A considerable number of subtypes of GABAergic interneurons diversify modes of cortical inhibition, enabling various types of information processing in the cortex. Thus, comprehensively understanding fate specification, circuit assembly, and physiological function of GABAergic interneurons is a key to elucidate the principles of cortical wiring and function. Recent advances in genetically encoded molecular tools have made a breakthrough to systematically study cortical circuitry at the molecular, cellular, circuit, and whole animal levels. However, the biggest obstacle to fully applying the power of these to analysis of GABAergic circuits was that there were no efficient and reliable methods to express them in subtypes of GABAergic interneurons. Here, I first summarize cortical interneuron diversity and current understanding of mechanisms, by which distinct classes of GABAergic interneurons are generated. I then review recent development in genetically encoded molecular tools for neural circuit research, and genetic targeting of GABAergic interneuron subtypes, particularly focusing on our recent effort to develop and characterize Cre/CreER knockin lines. Finally, I highlight recent success in genetic targeting of chandelier cells, the most unique and distinct GABAergic interneuron subtype, and discuss what kind of questions need to be addressed to understand development and function of cortical inhibitory circuits.

## INTRODUCTION

**FIGURE 1:**
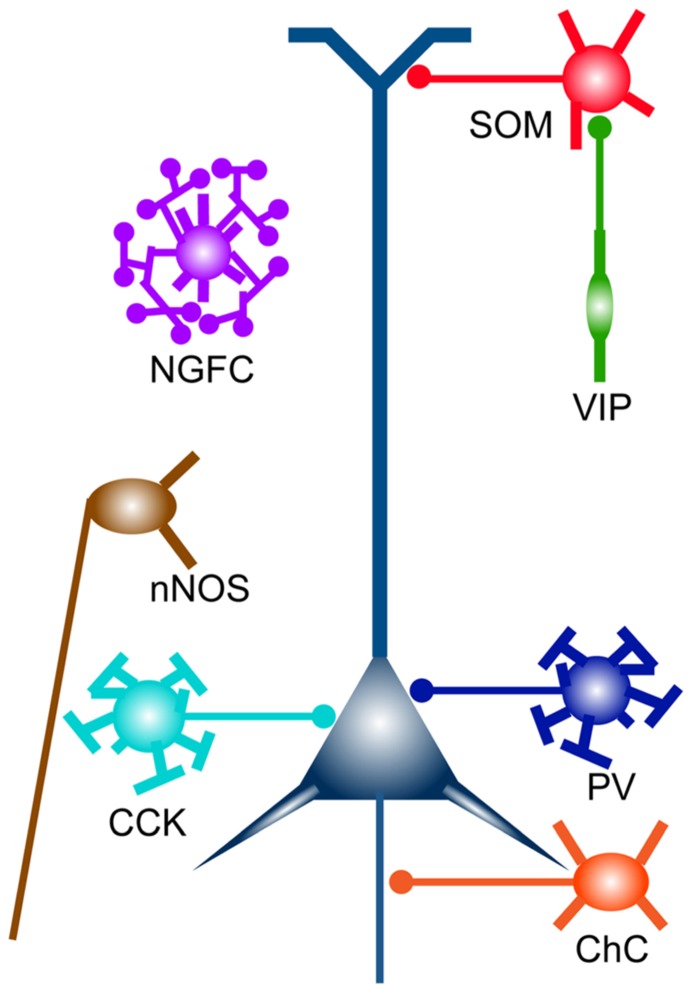
**Cortical GABAergic interneuron subtypes.** Broad subtypes of cortical GABAergic interneurons, which are characterized by gene expression and unique subcellular innervation. Distinct subcellular compartments of an excitatory pyramidal neuron (in indigo blue) are innervated by different classes of GABAergic interneurons (e.g., PV and CCK neurons, soma targeting; SOM neurons, dendrite targeting; ChCs, axon targeting). ChC, chandelier cell; NGFC, neurogliaform cell.

The mammalian neocortex is a highly evolved organ that plays a critical role in higher order brain functions such as perception, learning, memory, and behavioral outputs. It executes many kinds of neuronal computations through complex and delicate interactions between distinct types of neurons in the cortical and subcortical regions. Among many neuronal signals, excitatory and inhibitory neurotransmissions are the most fundamental components that shape activity patterns of neural networks. In particular, cortical inhibition locally provided by GABAergic interneurons has a key role in not merely overall balance of network excitability but also synaptic integration, spike timing, and synchrony of a neuronal ensemble (Isaacson and Scanziani, 2013). The various inhibitory regulations are mediated by diverse interneuron subtypes with unique physiological and morphological features (Markram et al., 2004; Klausberger and Somogyi, 2008). Subcellular compartment-specific innervation by distinct interneuron subpopulations [e.g., dendrite-, soma-, and axon initial segment (AIS)-targeting interneurons] also contributes to diversify neuronal computations (Somogyi et al., 1998; Buzsaki et al., 2004; **Figure 1**). Therefore, unraveling “subtype-specific” development, connectivity, and function of GABAergic interneurons will provide clues toward understanding how functional cortical circuits are wired and how the brain integrates information and generates outputs, which have been central issues in neuroscience.

In the past decade, an explosive development of genetic tools to investigate neuronal circuits molecularly, anatomically, and physiologically has been made. These tools allow us to obtain molecular expression profiles, identify connected neuronal populations, visualize signaling events and voltage changes, and optically manipulate neuronal activity (**Table 1**). In parallel, several methods to introduce these genetic tools into intact brains, including viral delivery, mouse genetics, and electroporation have been developed. Analysis methods such as new microscopy (e.g., super-resolution microscopy, high-throughput electron microscopy, and two photon microscopy) and high-throughput sequencing have also evolved, further enhancing the value of genetic probes and effectors. However, especially in the case of studies in GABAergic interneurons, these tools need to be expressed in specific subtypes for clear interpretations. Until recently, such targeted expression was not possible because there has been no systematic strategy that enables reliable expression of exogenous genes in GABAergic interneuron subtypes. This technical limitation has hampered a comprehensive understanding of development and function of GABAergic local circuits.

**Table 1:** Genetically encoded molecular tools for visualizing, measuring and manipulating neural circuits.

Molecular name	Purpose	Comments
mGRASP	Visualization of synapses	Split-GFP reconstitution, post-GRASP (neuroligin-based), post-GRASP (neuroligin-based)
FlaSh5	Measurement of voltage	Shaker potassium channel fused with GFP
SPARC	Measurement of voltage	Sodium channel fused with GFP
VSFP-butterfly1.2	Measurement of voltage	Voltage sensing domain of phospatase fused with mCitrine and mKate2
GCAMP6	Measurement of calcium level	A fusion of calmodulin, M13 domain of a myosin light chain kinase and GFP
SuperClomeleon	Measurement of chloride level	A fusion of CFP and YFP, chloride binding to YFP quenches the yellow fluorophore
EPAC	Measurement of cAMP level	epac1 fused with CFP and YFP
ChR2	Activation of neuronal activity	Light-induced non-selective cation channel
ChETA	Activation of neuronal activity	Light-induced non-selective cation channel
eNpHR	Inhibition of neuronal activity	Light-induced inward chloride pump
Arch	Inhibition of neuronal activity, measurement of voltage	Light-induced outward proton pump
M3 DREADD	Inducible activation of neuronal activity	Engineered G_q_-coupled muscarinic acetilcholine receptor induced by CNO
M4 DREADD	Inducible suppression of neuronal activity	Engineered G_i_-coupled muscarinic acetilcholine receptor induced by CNO
EGFP-L10a	Purification of translating mRNAs	Ribosomal protein L10a fused with GFP
HA-RPL22	purification of translating mRNAs	Ribosomal protein L22 tagged with HA epitope
myc-Argonaute2	Purification of microRNAs	Argonaute2 tagged with myc epitope
synaptopHluorin	Visualization of synaptic vesicle exocytosis and endocytosis	Synaptic vesicle protein fused with SEP
SEP-tagged membrane bound proteins	Visualization of membrane protein recycling	AMPA receptors have been fused with SEP
FRET-sensors for signaling proteins	Visualization of signaling events	FRET sensors for CAMKII, Ras, Rho, and Cdc42 have been developed

Genetic approaches exploited in model animals such as mice, fish, flies, and worms have provided the most powerful and reliable methods to dissect complex biological systems. The same concepts and techniques are applicable to neural circuit research, which demands highly specific manipulations such as cell type specific targeting. In this review, I will highlight our recent efforts to generate mouse lines targeting GABAergic interneuron progenitors and subtypes, which enable us to express genetically encoded sensors and effectors in specific groups of cortical interneurons. I will also discuss some remaining important questions to understand development and function of cortical interneuron subtypes, focusing on our recent progress in targeting chandelier cells (ChCs), the most distinct GABAergic interneuron subtype.

## GABAergic INTERNEURON SUBTYPES

To understand development, organization, and operation of GABAergic circuits, it is critical to take cellular diversity into account. Many lines of evidence have suggested that there are a myriad of GABAergic interneuron subtypes classified by physiology, morphology, connectivity, and gene expression (Markram et al., 2004; Klausberger and Somogyi, 2008; Rudy et al., 2010; **Figure 1**). Although researchers have tried to find a simple relationship between cell types distinguished by distinct criteria, one cell type defined by expression of a certain gene can exhibit several types of physiological properties, and vice versa. However, ultimately classification of GABAergic interneuron subtypes based on the expression of a combination of genes may reveal distinct cell types, since physiology and connectivity are likely explained by sets of genetic programs. Although it is still far from the stage that truly pure subtypes are described by gene sets, there are several markers that can delineate broad subclasses of GABAergic interneurons. Here I introduce major subtypes of GABAergic interneurons, which are classified by gene expression of calcium-binding proteins and neuropeptides, and representative minute cell types within each subtype.

### PARVALBUMIN NEURON

**FIGURE 2:**
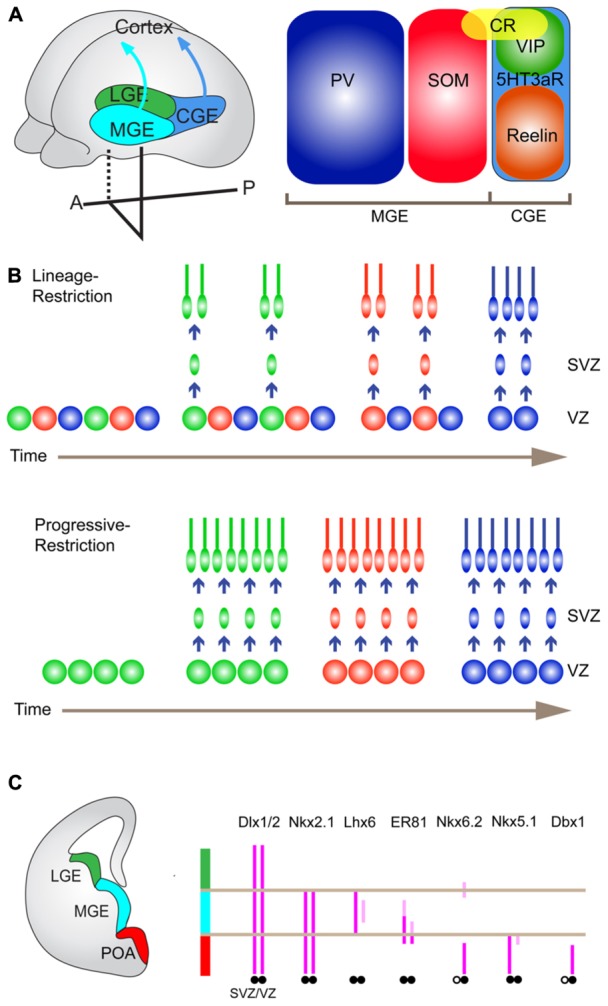
**Embryonic origins of cortical interneurons and their TF expression.** **(A)** Embryonic origins of cortical GABAergic interneurons (left) and three non-overlapping major interneuron subtypes (right). Cortical GABAergic interneurons are largely derived from the medial and caudal ganglionic eminence (MGE and CGE), and the preoptic area (POA), of the ventral forebrain. They tangentially migrate toward the cortex to establish cortical inhibitory local circuits. There are three distinct broad subtypes of GABAergic interneurons delineated by PV, SOM, and 5HT3aR. PV- and SOM-expressing neurons and 5HT3aR-expressing neurons are generated from the MGE and the CGE, respectively. **(B)** Potential mechanisms that generate cortical interneuron diversity. A lineage-restricted mechanism (upper). In this model, there coexist multiple types of progenitors, which are already determined to produce specific subtypes of interneurons with the fixed time schedule. A progressively restricted mechanism (lower). In this model, the fate potential of common progenitors changes over time to produce different subtypes of interneurons in a defined temporal order. **(C)** TFs that are expressed in distinct progenitor domains in the ventral forebrain.

PV is a calcium-binding protein, which is expressed in about 40% of total GABAergic interneurons in the somatosensory cortex (Fogarty et al., 2007; Lee et al., 2010; Xu et al., 2010b; **Figure 2A**). Most of PV-expressing interneurons are so called basket cells, which can be further subdivided by size of the cell body (e.g., large basket cell, small basket cell, and nest basket cell), and dendritic and axonal projection (Markram et al., 2004; Uematsu et al., 2008; Helmstaedter et al., 2009). Physiologically, PV-expressing basket cells are often fast-spiking (FS), characterized by a high-frequency train of action potentials (APs) with little adaptation (Kawaguchi et al., 1987; Cauli et al., 1997; Kawaguchi, 1997; Gibson et al., 1999; Xu and Callaway, 2009). It is widely accepted that PV basket neurons innervate the soma and proximal dendrites of excitatory pyramidal neurons (Martin et al., 1983; Gilbert, 1993; Kawaguchi and Kubota, 1997; **Figure 1**). Feedforward inhibition mediated through FS PV-expressing basket neurons can be found in several cortical networks including thalamocortical, translaminar, and interareal circuits (Shao and Burkhalter, 1996; Dantzker and Callaway, 2000; Porter et al., 2001; Pouille and Scanziani, 2001; Thomson et al., 2002; Dong et al., 2004; Gabernet et al., 2005). FS PV basket neurons strongly inhibit neighboring excitatory pyramidal neurons. It has been shown that PV basket neurons and pyramidal neurons that share common excitatory inputs tend to be reciprocally connected (feedback inhibition; Yoshimura et al., 2005). These connections may serve to regulate the precise time window in which the excitatory neurons can generate spikes in response to excitatory drives. In addition, thalamocortical and intracortical excitatory inputs onto FS PV basket neurons are depressed by high frequency stimulation, which mediates activity-dependent feedforward inhibition (Gabernet et al., 2005). PV-expressing basket cells also innervate other interneurons including other basket cells, and are electrically coupled with each other through gap junctions (Gibson et al., 1999; Tamas et al., 2000; Galarreta and Hestrin, 2002). It has been proposed that this feature may help to generate and maintain cortical network synchronization and oscillation (Tamas et al., 2000).

ChCs have been traditionally regarded as PV-expressing neurons although recent evidence has demonstrated that the majority of ChCs express no or little PV (DeFelipe et al., 1989; Taniguchi et al., 2013). ChCs are also FS neurons although their electrophysiological properties are slightly different from PV basket neurons (e.g., membrane time constant and input resistance; Woodruff et al., 2009; Taniguchi et al., 2013). ChCs form synapses specifically on AISs, which are sites of AP initiation, of excitatory pyramidal neurons (Somogyi, 1977). This striking morphological feature led researchers to propose a compelling hypothesis that ChCs powerfully control spike initiation, thereby synchronizing activity of a neuronal ensemble. Just recently, a systematic approach to studying this fascinating cell type has become possible due to the development of a novel genetic strategy (Taniguchi et al., 2013). Details will be described later.

### SOMATOSTATIN NEURON

Somatostatin (SOM) is a neuropeptide, which is expressed by 30% of cortical interneurons. SOM-expressing interneurons have little overlap with PV-expressing interneurons (Fogarty et al., 2007; Lee et al., 2010; Xu et al., 2010b; **Figure 2A**). It is widely accepted that SOM neurons are classified as dendrite-targeting cells with respect to the output connectivity (Karube et al., 2004; Dumitriu et al., 2007; **Figure 1**). Representative cell types that belong to SOM-expressing neurons include Martinotti cells in the neocortex, which project ascending axons that horizontally bifurcate in layer 1 (L1; Kawaguchi and Kubota, 1997; Wang et al., 2004). Martinotti cells form synapses onto the apical tufts of pyramidal cell dendrites (Kawaguchi and Kubota, 1997; Wang et al., 2004). In the cortex, Martinotti cells are distributed abundantly in L5 but are also found in other layers except for L1 (Kawaguchi and Kubota, 1997; Wang et al., 2004). From the electrophysiological point of view, they are categorized as adapting regular-spiking non-pyramidal cells, or burst spiking non-pyramidal cells (Kawaguchi and Kubota, 1997; Gibson et al., 1999; Xu et al., 2006; Miyoshi et al., 2007). In contrast to PV neurons, Martinotti cells receive excitatory synapses that are strongly facilitating, which drive their feedback or feedforward inhibition to pyramidal neurons in an activity-dependent manner (Kapfer et al., 2007; Silberberg and Markram, 2007; Fanselow et al., 2008; Hull et al., 2009). Due to such a dynamic input property, Martinotti cell-mediated inhibition can be more efficient when the network activity is increased (Kapfer et al., 2007; Silberberg and Markram, 2007). Therefore, it is conceivable that the shift from somatic inhibition by PV neurons to dendritic inhibition by Martinotti cells occurs as the circuit activity increases. Such a cellular mechanism may be utilized to shift the inhibitory impact on pyramidal neuron activity from gating control to local synaptic integration in an activity-dependent fashion.

Martinotti cells can be further divided into two subclasses defined by the presence or absence of calretinin (CR), the calcium-binding protein. SOM^+^/CR^+^ and SOM^+^/CR^-^ Martinotti cells display differences in not only dendritic organization but also input connectivity (Xu et al., 2006). In L2/3, SOM^+^/CR^+^ cells are innervated by L2/3 pyramidal neurons, whereas SOM^+^/CR^-^ cells receive excitatory synaptic inputs from both L2/3 and L4 pyramidal neurons (Kapfer et al., 2007; Silberberg and Markram, 2007). Another type of cortical SOM neuron, anatomically and physiologically distinct from Martinotti cells is the X94 cell (Ma et al., 2006). X94 is the name of the transgenic mice in which green fluorescent protein (GFP) is expressed in a subpopulation of SOM neurons under the control of the GAD67 promoter. X94 cells are found in L4 and L5, and heavily innervate L4 cells (Ma et al., 2006).

### 5HT3aR NEURON

The 5HT3a receptor (5HT3aR) is the inotropic serotonergic receptor, which is expressed in most, if not all, GABAergic interneurons that express neither PV nor SOM and comprise about 30% of total cortical interneurons (Lee et al., 2010; **Figure 2A**). 5HT3aR neurons are very heterogeneous physiologically, anatomically, and biochemically but roughly divided into two subclasses, vasoactive intestinal peptide (VIP) neurons (see below) and non-VIP neurons (Lee et al., 2010). VIP neurons compose around 40% of the 5HT3aR population in the somatosensory cortex (**Figure 2A**). Non-VIP 5HT3aR neurons corresponding to 60% of the 5HT3aR neurons, include Reelin-expressing neurons. The Reelin-positive neurons are a primary population (more than 80%) of non-VIP 5HT3aR cells (Lee et al., 2010; **Figure 2A**). A fraction of the Reelin^+^/VIP^-^/5HT3aR^+^ neurons contains neurogliaform cells (NGFCs), which have small and round somata with a dense axonal plexus containing small presynaptic boutons (Kawaguchi and Kubota, 1997; Olah et al., 2007; Lee et al., 2010). NGFCs show unique properties regarding their connectivity and neurotransmission. They form gap junctions not only with other NGFCs but also with other classes of GABAergic interneurons, implicating roles in generating synchronized activity and recruiting distinct inhibitory modes driven by different classes of GABAergic interneurons (Price et al., 2005; Simon et al., 2005; Zsiros and Maccaferri, 2005). The synapses formed by NGFCs are remarkably different from those formed by other interneurons in that they have unusually wide synaptic clefts and sometimes no obvious postsynaptic structures (Olah et al., 2009; **Figure 1**). NGFCs can cause slow and sustained inhibitory postsynaptic potentials (IPSPs) in target neurons within the area covered by their axonal plexus, through the concomitant activation of slow GABA_A_ and GABA_B_ receptors (Tamas et al., 2003; Olah et al., 2007). These anatomical and physiological observations suggest that NGFCs exert inhibitory effects on remote target neurons (non-synaptic partners) by diffusion through the extracellular fluid of neurotransmitters released from their axon terminals, which is known as volume transmission (Olah et al., 2009). It has been shown that 5HT3aR neurons can be activated by serotonin and acetylcholine, which are released by subcortical long-range neuromodulatory axons (Ferezou et al., 2002; Lee et al., 2010). These neuromodulators increase the membrane potential of 5HT3aR neurons, thus making them more excitable. Therefore, neuromodulators may open the gate to drive the feedforward or feedback inhibition by 5HT3aR neurons.

### VASOACTIVE INTESTINAL PEPTIDE (VIP) NEURON

As described above, the neuropeptide VIP is expressed in a subset of interneurons that does not overlap with SOM and PV neurons (Lee et al., 2010; Rudy et al., 2010; **Figure 2A**). A significant number of VIP neurons coexpress CR and display bitufted/bipolar morphology (Cauli et al., 2000; Caputi et al., 2009; Lee et al., 2010; Miyoshi et al., 2010; **Figure 2A**). By physiological characterizations, these neurons are usually referred to as irregular-spiking cells (Cauli et al., 1997, 2000; Porter et al., 1998; Ferezou et al., 2002; Miyoshi et al., 2010). The second major subtype of VIP neurons displays bitufted/multipolar morphology but does not express CR (Lee et al., 2010). These neurons are referred to as fast-adapting cells, which show rapidly adapting firing traits (Lee et al., 2010; Miyoshi et al., 2010). It was reported that VIP neurons form synapses on dendritic spines and shafts of pyramidal neurons (Kawaguchi and Kubota, 1996; Kawaguchi and Kubota, 1997), and some of them appear to preferentially innervate other interneurons (Acsady et al., 1996; David et al., 2007). However, recent evidence has indicated that VIP neurons inhibit pyramidal neurons very little and exert inhibitory effects on SOM neurons selectively (Pfeffer et al., 2013; **Figure 1**). These results may suggest that VIP neurons disinhibit pyramidal neurons. On the other hand, as SOM neurons inhibit PV neurons as one of their postsynaptic targets (Pfeffer et al., 2013), activation of VIP cells may also lead to more PV cell firing and increase perisomatic inhibition in pyramidal neurons.

### CHOLECYSTOKININ (CCK) NEURON

In addition to PV-expressing basket cells, the neuropeptide CCK-expressing neurons comprise the other class of basket cells (**Figure 1**). Just like PV neurons, CCK basket cells are thought to control the phasing and synchronization of neural ensembles (Freund and Katona, 2007). However, CCK basket cells have unique molecular and physiological features, which are different from those of PV basket cells. For instance, GABAergic neurotransmission by CCK basket cells is mediated through α2-containing GABAa receptors, which show slow kinetics whereas PV basket cells utilize α1-containing GABAa receptors, which mediate fast GABAergic responses at postsynaptic sites (Nyiri et al., 2001; Freund and Katona, 2007). Another noteworthy property of CCK basket cells is a plastic change in transmitter release in response to retrograde signals. Presynaptic terminals of CCK neurons express the cannabinoid type 1 (CB1) receptors, which are activated by endogenous cannabinoids released from postsynaptic pyramidal neurons (Katona et al., 1999). The activation of CB1 receptors leads to a reduction in GABA release (Wilson and Nicoll, 2001; Bodor et al., 2005; Hill et al., 2007; Galarreta et al., 2008).

### NEURONAL ISOFORM OF NITRIC OXIDE SYNTHASE (NNOS) NEURON

Nitric oxide synthesized by nitric oxide synthase (NOS) is a retrograde signaling molecule that modulates neuronal transmission, although its roles in GABAergic interneurons are largely unknown. nNOS is broadly expressed in the cerebral cortex during development (Bredt and Snyder, 1994) but is confined to a subpopulation of GABAergic interneurons (Kubota et al., 2011). In the hippocampus, this class of neurons includes NGFCs and ivy cells (Fuentealba et al., 2008). In the neocortex, nNOS-expressing interneurons are classified into two types, type I and type II. Type I neurons represent an unusual population of GABAergic interneurons that project long-range axons ipsi- and contralaterally within the cortex and to subcortical areas (Tomioka et al., 2005; Higo et al., 2009; **Figure 1**). Notably, type I neurons selectively spike during slow wave sleep when most cortical neurons are relatively silent (Kilduff et al., 2011). These features suggest that type I neurons may coordinate the activity of different brain areas which are apart from each other. Type II neurons appear to include NGFCs, some of which also express Reelin and 5HT3aR as described above (Lee et al., 2010).

## ORIGINS AND SUBTYPE SPECIFICATION OF GABAergic INTERNEURONS

In the nervous system, the diversification of neuronal cell types is a common strategy to ensure functional complexity and flexibility of the neuronal networks. Cortical GABAergic interneurons display the highest degree of heterogeneity, which brings up a lot of important and exciting questions but has hampered fine experiments needed to gain definitive conclusions. Understanding cellular and molecular mechanisms that generate diverse subtypes of GABAergic interneurons is critical, not only for unraveling mechanisms for the subsequent assembly of neural circuits, but also for developing genetic methods to visualize and manipulate distinct subtypes at any stage from birth to functional maturation. Although our knowledge on mechanisms for subtype specification of GABAergic interneurons is still very limited, there is highly suggestive information regarding cell fate determination mainly coming from other systems such as motor-related neurons in vertebrates, neurogenesis of cortical excitatory neurons, and neurogenesis in fruit flies. In particular, because of their relatively simple cellular organization, as well as a rich repertoire of molecular markers that specifically label neuronal subtypes, the motor-related neurons in the spinal cord have provided ideal experimental systems to study cell type specification (Tanabe and Jessell, 1996; Arber, 2012). What one could learn from motor-related neurons is as follows: (1) inductive signals secreted from local groups of neuronal and non-neuronal cells pattern neuroepithelium along the dorsoventral and rostrocaudal axes to create distinct progenitor domains (Jessell, 2000; Dasen et al., 2003); (2) at the same dorsoventral and rostrocaudal positions, local interactions between different neuronal populations, such as early-born neurons and late-born neurons provide them with discrete identities (Sockanathan and Jessell, 1998); (3) the combinatorial expression of transcription factors (TFs) including Hox proteins and LIM-homeodomain TFs in postmitotic neurons define neuronal identities such as connectivity (Kania and Jessell, 2003; Dasen et al., 2005). Complimentarily to these influential findings, recent studies have proposed two mechanisms (lineage-restriction versus progressive-restriction), by which different cell types are generated from progenitors in the same mitotic domain (Franco and Muller, 2013). Radial glial cells (RGCs), which reside in the ventricular zone (VZ) of the cortex, are progenitors for excitatory pyramidal neurons in L2 through L6 (Franco and Muller, 2013). Recent elegant experiments using genetic lineage-tracing techniques have demonstrated that Cux2^+^ (cut-like homeobox 2 protein) RGCs produce later-born upper-layer neurons, while earlier-born lower-layer neurons are generated from Cux2^-^ RGCs (Franco et al., 2012). Cux2^+^ RGCs coexist with Cux2^-^ RGCs even during earlier-born lower-layer neurogenesis. Neurons that are prematurely born from Cux2^+^ RGCs by the forced exit from the proliferative states at this earlier stage show no change in their normal laminar position and molecular profiles (Franco et al., 2012). These results indicate that at least in a fraction of cortical pyramidal neurons the fate decision occurs in a cell-lineage-dependent manner rather than a birth date-dependent manner (lineage-restriction). In other words, a neuronal progenitor is intrinsically programmed to generate a specific type of neuron regardless of birth timing (**Figure 2B**). The other mechanism that explains production of different neuronal types is temporal restriction of competence of a common progenitor to differentiate into certain cell types (Franco and Muller, 2013). This concept was established by studies in fruit fly neurogenesis, in which the same neuroblast progressively gives rise to different neuronal subtypes by sequentially changing the expression of TFs such as hunchback, Krupple, Pdm, and Castor (progressive-restriction; Isshiki et al., 2001; Pearson and Doe, 2003; **Figure 2B**). Taken together, strategies to diversify neuronal cell types include formation of distinct progenitor domains, lineage-dependent cell fate determination, progressive changes in cellular competence to give rise to certain cell types, and intrinsic/extrinsic controls in postmitotic neurons to accomplish specific terminal differentiation. At present it still remains unclear to what extent GABAergic interneurons utilize these strategies to generate a variety of cell types, but it is likely that all the possibilities are used in combination. Below I will outline our current understanding in origins of GABAergic interneurons and molecular determinants for subtype specification.

### ORIGINS OF GABAergic INTERNEURONS

Most, if not all, cortical GABAergic interneurons are generated from the ventral telencephalon including the medial ganglionic eminence (MGE), the preoptic area (POA), and the caudal ganglionic eminence (CGE), in non-primate mammals. Immature GABAergic interneurons tangentially migrate toward the cortex (Gelman and Marin, 2010; **Figure 2A**).

The MGE produces about 50–60% of cortical interneurons in mice, most of which are PV and SOM neurons (Xu et al., 2004; Butt et al., 2005; Wonders and Anderson, 2006; Fogarty et al., 2007; Miyoshi et al., 2007; **Figure 2A**). How different classes of interneurons are produced within the MGE is largely unknown but recent studies have demonstrated that each subtype of interneuron has preferential spatial and temporal origins. The systematic *in situ* hybridization analysis for many TFs expressed in the MGE identified five distinct progenitor domains called pMGE1 to pMGE5. Each of these domains expresses a unique combination of genes, and led to the hypothesis that each progenitor domain generates different subtypes of GABAergic interneurons (Flames et al., 2007). Consistent with this view, classical transplantation experiments, in which the MGE was dissected into three pieces (dorsal, medial, and ventral) and cells from each piece were injected into wild type host brains, indicated that the dorsal and ventral division preferentially gives rise to SOM- and PV-expressing neurons, respectively (Wonders et al., 2008). Furthermore, genetic fate mapping of the dorsal MGE progenitors expressing Nkx6.2 showed that this domain predominantly produces Martinotti cells, which are double positive for SOM and CR (Fogarty et al., 2007; Sousa et al., 2009; **Figures 2A,C**). Moreover, it has been shown that the sonic hedgehog (Shh) signaling effectors such as Gli1, Gli2, Ptch1, and Nkx6.2 are enriched in the dorsal MGE (Flames et al., 2007; Wonders et al., 2008; Yu et al., 2009) and a higher level of Shh signaling promotes the production of SOM neurons at the expense of PV neurons (Xu et al., 2010a). In addition to the spatial patterning of progenitor domains, conditional genetic-fate mapping of MGE progenitors at different time points correlated the birthdates with specific features of GABAergic interneurons (Miyoshi et al., 2007). For example, earlier-born and later-born neurons are positioned in deeper and more superficial layers in the cortex, respectively (Miyoshi et al., 2007). It is also reported that more SOM neurons are produced at earlier time points (Miyoshi et al., 2007). These results suggest two possible regulations at a progenitor level, that is, lineage-restriction and progressive restriction (**Figure 2B**). More recent results based on the clonal analysis have demonstrated that clonal neurons are born isochronically and typically form a cluster within one or two neighboring layers rather than in a columnar manner, favoring the presence of lineage-restricted progenitors (Ciceri et al., 2013).

Recent work has demonstrated that approximately 10% of total cortical GABAergic interneurons are derived from POA progenitors (Gelman et al., 2009, 2011). Just like the MGE, the POA is molecularly divided into two distinct progenitor domains, the POA1 and the POA2 (Flames et al., 2007). Nkx5.1 appears to be expressed in a subpopulation of progenitors in the POA1 (**Figure 2C**). Genetic fate mapping experiments using Nkx5.1-Cre mice revealed that roughly one third of the progenies derived from these progenitors express neuropeptide Y (NPY) and none of them express representative interneuron markers such as PV, CR, SOM, VIP, and nNOS (Gelman et al., 2009). In a complementary manner, Dbx1 is expressed in progenitor cells in the POA2, which is located right ventral to the POA1 (Gelman et al., 2011; **Figure 2C**). A genetic fate mapping study with Dbx1-Cre mice indicated that Dbx1-expressing progenitors in the POA2 produce major subtypes of cortical GABAergic interneurons including PV, SOM, Reelin, CR, NOS, and VIP neurons, which share neurochemical properties with those derived from the MGE or the CGE (Gelman et al., 2011). The immunohistochemical analysis showed that the POA derived PV and SOM neurons express little Lhx6, which is expressed by those derived from the MGE, suggesting two functionally distinct populations within PV and SOM neurons (Gelman et al., 2009, 2011).

CGE has been anatomically defined as a posterior extension of the lateral ganglionic eminence (LGE) and the MGE with ambiguity. However, recent studies have demonstrated that the CGE can be molecularly delineated with a unique set of genes (i.e., Couptf1, Couptf2, Prox1, and Mash1), and divided into several progenitor domains (Flames et al., 2007; Kanatani et al., 2008; Willi-Monnerat et al., 2008; Long et al., 2009). What was suggested by classical transplantation experiments and DiI labeling, and subsequently validated by genetic fate mapping experiments using Mash1-CreER BAC transgenic lines was that the CGE produces around 30% of all cortical interneurons, which express 5HT3aR and most of which display bipolar and bitufted morphologies (Anderson et al., 1997, 2001; Nery et al., 2002; Butt et al., 2005; Lee et al., 2010; Miyoshi et al., 2010; **Figure 2A**). As mentioned in the previous section, 5HT3aR-positive neurons express different combinations of CR, VIP, NPY, and Reelin (Lee et al., 2010).

### TRANSCRIPTIONAL REGULATION OF GABAergic INTERNEURON SPECIFICATION

Exploration of the transcriptional regulation of GABAergic interneuron specification and diversification has begun to be explored, but many questions still remain. The first step toward production of GABAergic interneurons is to divide the neuroepithelium into the pallium (the dorsal telencephalon that generates cortical pyramidal neurons) and the subpallium (the ventral telencephalon that generates cortical interneurons). Upon patterning of the neuroepithelium along the dorsal–ventral axis through the actions of morphogens such as Shh (Fuccillo et al., 2004; Xu et al., 2005) and bone morphogenetic proteins (Lee and Jessell, 1999; Solloway and Robertson, 1999), different sets of TFs begin to be expressed in distinct progenitor domains. Pax6 and Gsh2 are expressed in the pallium and the dorsal subpallium, respectively, and play a key role in specification and maintenance of these territories by well-characterized cross-repressive interactions (Yun et al., 2001). A ventral part of the subpallium corresponding to the MGE and the POA is delineated by the expression of Nkx2.1 (**Figure 2C**), which is induced and maintained by the activity of Shh (Chiang et al., 1996; Anderson et al., 2001; Fuccillo et al., 2004; Xu et al., 2004, 2005).

A loss-of-function study revealed that in Nkx2.1 null mutant mice, the MGE is mis-specified to the fate of the LGE and as a consequence, more than half of the cortical interneurons including SOM-, NPY-, and calbindin-expressing neurons are lost (Sussel et al., 1999). Since Nkx2.1 knockout mice die at birth due to defects in other organs (Sussel et al., 1999), this study is unable to clarify what types of interneurons are lost in the mature cortex and whether Nkx2.1 plays an essential role in cell fate specification in addition to patterning of the MGE. A subsequent study utilizing a conditional Nkx2.1 allele demonstrated that removal of Nkx2.1 gene function after establishment of MGE identity alters the fate of MGE-derived progenitors so that VIP/CR-expressing neurons, which normally originate from the CGE, are generated instead of MGE-derived PV- and SOM-expressing neurons (Butt et al., 2008). These results indicate that Nkx2.1 functions as a molecular switch that favors fates of MGE progenitors rather than those of LGE and CGE.

Lhx6, the LIM homeodomain TF, is a direct downstream target of Nkx2.1 (Du et al., 2008). This gene is expressed in intermediate progenitors in the subventricular zone (SVZ) of the MGE (Sussel et al., 1999; Flames et al., 2007; Du et al., 2008) and continues to be expressed in developing and mature postmitotic cortical interneurons (**Figure 2C**). In the mature cortex, the expression of Lhx6 is confined to PV- and SOM-expressing neurons, which are produced exclusively in the MGE (Fogarty et al., 2007; Liodis et al., 2007; Zhao et al., 2008b). Studies of Lhx6 null mutant mice found two major phenotypes, namely, cell migration and cell type specification defects. First, Lhx6 deficient neurons show a delay in arriving at the cortex from the MGE and defects in their proper laminar positioning (Liodis et al., 2007; Zhao et al., 2008b). Consistent with these findings, the expression of several receptor molecules such as CXCR4, CXCR7, and ErbB4, which are involved in interneuron migration and positioning, is reduced (Zhao et al., 2008b). Second, they fail to develop the expression of PV and SOM, either of which is expressed in normal Lhx6-expressing neurons (Liodis et al., 2007; Zhao et al., 2008b). Additional studies that identify and functionally characterize the downstream cascade of Lhx6 have provided hints as to how Lhx6 controls cortical interneuron diversity.

Sox6, a Sry-related HMG-box containing TF, is expressed in most immature and mature cortical interneurons that originate from the MGE (Azim et al., 2009; Batista-Brito et al., 2009). In Lhx6 null mutant mice, MGE-derived interneurons lose the expression of Sox6, suggesting that Lhx6 activity is required for maintenance of Sox6 expression (Batista-Brito et al., 2009; Flandin et al., 2011). In the mature cortex of Sox6 null and conditional mutants, the expression of PV and SOM is reduced in MGE-derived interneurons and their laminar position is significantly altered (Azim et al., 2009; Batista-Brito et al., 2009). Electrophysiological analysis of Sox6 mutant interneurons indicated that basket cells which normally express PV have characteristic but immature fast-spiking intrinsic properties (Batista-Brito et al., 2009). These results suggest that Sox6 is necessary for proper positioning and maturation but not specification of the MGE-derived interneurons.

During neurogenesis of cortical interneurons, Shh is expressed not only in progenitors in the VZ of the ventral subpallium but also in earlier-born interneurons in the mantle zone (MZ) of the MGE (Flandin et al., 2011). Genetic and molecular biological analyses have revealed that the expression of Shh in the MZ is controlled by redundant activities of Lhx6 and Lhx8, which can bind to and regulate the Shh enhancer (Flandin et al., 2011). Genetic deletion of Shh in earlier born interneurons in the MZ leads to reduced Shh signaling in the overlying rostrodorsal MGE progenitor zone, which in turn results in downregulation of Lhx6, Lhx8, and Nkx2.1 (Flandin et al., 2011). Such transcriptional alterations in this mutant apparently cause defects in the production of late-born PV and SOM-expressing MGE-derived interneurons (Flandin et al., 2011). These results indicate that one mechanism, by which different types of cortical interneurons are produced, is a cell non-autonomous effect of Lhx6 and Lhx8 activities in the MZ on progenitors in the MGE, which is mediated through the Shh activity.

The Dlx homeobox genes (Dlx1, Dlx2, Dlx5, and Dlx6) are widely expressed in subpallial progenitors of the LGE, MGE, and CGE (Eisenstat et al., 1999), and continue to be expressed in most of their postmitotic progenies in embryonic, postnatal, and mature cortices (Cobos et al., 2007; **Figure 2C**). Cortical interneurons in Dlx1/2 and Dlx5/6 double mutants show common defects in tangential migration (Anderson et al., 1997; Panganiban and Rubenstein, 2002; Wang et al., 2010). However, recent studies have shown that different members of Dlx genes have unique gene expression dynamics and specific functions throughout development and maturation. For instance, it has been shown with transplantation experiments that deletion of Dlx5 or Dlx5/6 genes specifically reduces the number of PV-expressing neurons in the mature cortex and causes abnormal dendritic branching (Wang et al., 2010). In the adult cortex, Dlx1 is detected preferentially in SOM- and CR-expressing neurons (Cobos et al., 2007). Consistent with the expression pattern in the mature cortex, Dlx1 knockout mice exhibit specific and progressive loss of SOM- and CR-expressing cortical interneurons due to apoptotic cell death and immature dendritic arborization in these classes of interneurons (Cobos et al., 2007).

Transcriptional mechanisms that control the fate determination of the CGE have remained largely elusive. Gsh2 is a homeodomain TF that is normally expressed in the dorsal LGE and the CGE (Hsieh-Li et al., 1995). Conditional removal and ectopic expression of a Gsh2 gene revealed that Gsh2 plays a role in generation of CGE-derived CR-expressing bipolar interneurons (Xu et al., 2010a). Couptf1 is an orphan nuclear receptor, whose expression becomes gradually restricted to the CGE by midgestation (Lodato et al., 2011). Conditional inactivation of Couptf1 in intermediate progenitors and postmitotic neuroblasts in the CGE leads to a reduction in the number of late-born VIP- and CR-expressing bipolar neurons together with the concurrent increase in the number of PV-expressing neurons, without changing the total number of cortical GABAergic interneurons (Lodato et al., 2011). The results obtained from these studies provide clues regarding the transcriptional codes that determine cortical interneuron subtypes that are derived from the CGE.

## GENETIC TECHNIQUES TO SPY ON STRUCTURE AND FUNCTION OF NEURAL CIRCUITS

Recent advances in molecular tools as well as genetic strategies to interrogate structure and function of neural circuits hold promise for dramatically improving our understanding of brain function. Here I briefly summarize essential genetic techniques to investigate neural circuits along with currently available molecular tools (**Table 1**). For more details regarding these techniques, other reviews should be referred.

### VISUALIZING NEURONAL STRUCTURE AND CONNECTIVITY

Visualization of neurons is the first step toward understanding the developmental assembly and organization of neural circuits. Fluorescent proteins (XFPs) have been used to visualize neuronal structures both in live and fixed tissues (Shaner et al., 2005; Luo et al., 2008). Multi-color labeling facilitates the investigation of neuronal connectivity, and dynamic interactions between axons and dendrites. However, traditional methods relying on one or two XFPs are not enough to describe complex neural networks that consist of a large number of cell bodies, axons, and dendrites. A genetic method called “Brainbow” dramatically increases the number of colors used for labeling individual neurons, thereby enabling clear separation of neighboring cells and processes in the crowded neuropil at high resolution. This method uses Brainbow mice, which have tandem repeats of a transgene containing three or four XFPs at a single genomic site (Livet et al., 2007; Cai et al., 2013). When crossed with an appropriate Cre driver mouse, Cre-loxP recombination induces stochastic expression of a single XFP from transgenes. As a consequence, individual neurons have different ratios of XFPs, generating approximately 100 different colors. A potential limitation of Brainbow is that the expression of transgenes may not be universal since Brainbow mice are generated with a transgenic approach and utilize a Thy-1 promoter whose activity is restricted in mature neurons and not ubiquitous across neuronal types. This problem can be resolved by creating a knockin mouse, which has multiple Brainbow transgenes together with a ubiquitous promoter in a constitutively active locus such as ROSA26.

Gaining a wiring diagram of the brain helps to predict and understand how the brain works. Traditional methods such as electron microscopy, paired recordings, and dye-filling have pioneered this field (Kawaguchi and Kubota, 1997) but these approaches are not efficient enough to rapidly and systematically collect data. Recently, novel genetic methods used to visualize bona fide connected neuron pairs have revolutionized how we investigate neuronal connectivity. Retrograde mono-trans-synaptic labeling based on recombinant rabies viruses (RVs) visualizes input neurons that are directly connected to defined starter neurons (Wickersham et al., 2007). In the genome of recombinant RV, a gene encoding rabies envelope glycoprotein (RG) is swapped with the enhanced GFP *(egfp)* gene. Since RG is involved in the assembly of viral particles and the trans-synaptic transportation of the viruses, recombinant RVs cannot spread to presynaptic input cells without exogenously supplied RG. Therefore, when starter neurons express RG, recombinant RVs can spread only once to presynaptic input cells but are unable to spread beyond direct input cells. To achieve selective infection in defined starter cells, recombinant RVs are pseudotyped with the envelope glycoprotein (EnvA) of an avian virus. Pseudotyped recombinant RVs specifically infect starter cells expressing the EnvA receptor, TVA. Thus, when starter cells express both RG and TVA, and pseudotyped recombinant RVs infect these cells, direct input neurons are labeled with EGFP. The drawbacks of this method are (1) underestimated numbers of input neurons due to incomplete labeling caused by unknown reasons, and (2) toxicity that is observed approximately 10 days after infection. Nevertheless, this viral strategy is currently the most powerful approach to identify connected neurons at the light microscopy level.

Another recently developed approach to dissect neural connectivity is the use of split GFPs. Split GFPs are two fragments that together comprise the entire GFP. These fragments are separately fused to a pair of proteins that are known to interact. When the fusion proteins associate with each other the chromophore of GFP is reconstituted, generating fluorescence. This principle was first applied to detect synaptic connections in *Caenorhabditis elegans*, and is called GFP reconstitution across synaptic partners (GRASPs; Feinberg et al., 2008). The GRASP was further adjusted to the mammalian nervous system, which established the mammalian GRASP (mGRASP; Kim et al., 2011). In the mGRASP method, neurexin and neuroligin, which are trans-synaptically interacting proteins, are used as a presynaptic and postsynaptic carrier of the split GFP fragment, respectively. One potential pitfall of the GRASP is that the interaction of the reconstituted GFP between pre- and postsynaptic carriers may be too strong, thus causing irreversible synaptic contacts. Therefore, although the mGRASP provides a powerful tool to illuminate existing synapses, it remains unclear whether this method is suitable to observe dynamic events such as synapse formation and elimination during development.

### MEASURING NEURONAL ACTIVITY

Recording activity from a neuronal ensemble is crucial for elucidating how information is represented in the brain and how this activity is generated through interactions between multiple inputs. Genetically encoded sensors of neuronal activity and modulation have been developed and have provided valuable insights into principles of neuronal processing (Peterka et al., 2011).

Voltage sensors allow a direct observation of membrane potential including subthreshold excitatory and inhibitory responses in a non-invasive manner bypassing the need for electrodes. Voltage-dependent conformational changes in the voltage-sensing domain lead to either changes in the intensity of the fluorescent protein or changes in Forster resonant energy transfer (FRET) between fluorescent proteins. Prototypic genetically encoded voltage indicators were constructed simply by fusing a fluorescent protein with a voltage-sensing domain of an ion channel (e.g., FlaSh5 and SPARC; Siegel and Isacoff, 1997; Baker et al., 2007). However, signal changes in fluorescence were too small to reliably detect voltage changes with these indicators (<5% per 100 mV; Peterka et al., 2011). In addition, the kinetics of these reporters are generally too slow to capture action potentials (Peterka et al., 2011). These problems have recently been improved in VSFP-Butterfly 1.2, which is composed of a voltage-sensing domain of voltage-activated phosphatase and fluorescent proteins, mCitrine and mKate2 (Akemann et al., 2013). The voltage change-induced conformational change of VFSP-Butterfly 1.2 gives rise to FRET between mCitrine and mKate2 (22% per 100 mV; Akemann et al., 2013). Curiously, Arch (archaerhodopsin-3) was originally used for neuronal silencing but can also work as a voltage sensor (Kralj et al., 2013). At least *in vitro*, Arch exhibits excellent temporal resolution that distinguishes between single action potentials (Kralj et al., 2013). More optimization of these genetically encoded voltage sensors will be necessary to advance “optophysiology” in the near future.

Calcium sensors are now widely used both *in vivo* and *in vitro* to measure neuronal activity as an alternative method to voltage sensors. To date, the most popular genetically encoded calcium indicator is GCaMP (e.g., GCaMP3, GCaMP5, and GCaMP6), consisting of calmodulin (CaM), which contains a calcium-binding site, the M13 peptide, which binds to the calcium-bound form of CaM, and a circulary permuted EGFP (Tian et al., 2009; Akerboom et al., 2012; Chen et al., 2012). The action potential firing causes calcium influx into neurons through voltage-gated calcium channels. Upon calcium binding, CaM undergoes a conformational change and then forms a complex with the M13 peptide, resulting in an increase in the fluorescence of EGFP. It has been shown that fluorescent signals measured from GCaMP6 can detect single action potentials both *in vitro* and *in vivo* (Chen et al., 2012). One common concern about calcium sensors is the calcium buffering effect, which may disrupt natural biological conditions.

Imaging intracellular chloride ion concentration is useful for revealing spatiotemporal dynamics of inhibitory regulation in the neural network. Clomeleon is a genetically encoded chloride ion indicator, which is composed of cyan fluorescent protein (CFP) and yellow fluorescent protein (YFP) linked by a flexible 24 amino acid linker (Kuner and Augustine, 2000). Without chloride ions, Clomeleon shows FRET between CFP and YFP due to the close proximity of the two fluorophores. Interestingly, the YFP molecule contains a chloride-binding site. Thus, when chloride binds to the site, yellow fluorescence is quenched and FRET is reduced. A major problem of the current version of Clomeleon is the low affinity for a chloride ion. This means that chloride concentrations must be increased above the physiological range in order for Clomeleon to detect changes in chloride influx. Fortunately, a new version of Clomeleon called SuperClomeleon has been shown to work in physiological conditions due to a number of improvements, including an increased affinity for chloride ions (Grimley et al., 2013).

Neuromodulation has a significant impact on neuronal processing in the normal brain and has been implicated in the pathogenesis of several psychiatric diseases (Arnsten et al., 2012). In particular, dopaminergic control plays a key role in motor control, motivation, and cognition (Tritsch and Sabatini, 2012). Monitoring dopamine signaling is useful for understanding the spatiotemporal dynamics of neuromodulation in a neuron or a neuronal ensemble. The activation of dopamine receptors leads to alterations in cAMP concentration (Tritsch and Sabatini, 2012). Thus, Epac-based FRET sensors, which contain a part of the cAMP-binding protein Epac1 flanked by CFP and YFP, can be used to detect dopamine signaling (van der Krogt et al., 2008). Upon cAMP binding, a conformational change occurs in Epac, which causes a decrease in FRET (van der Krogt et al., 2008). Several modifications to the original Epac sensor have been made to improve dynamic range, signal-to-noise ratio and photostability.

### MANIPULATING NEURONAL ACTIVITY

Rapid bidirectional manipulations of neural activity are required for providing evidence for the causal relationship between spike patterns and behavior/brain function. Recent developments in optogenetic tools has enabled optical control of neural circuits by driving or inhibiting neuronal spikes with light. Channelrhodopsin-2 (ChR2) is a non-selective cation channel that is opened by blue light (Nagel et al., 2003). When expressed in the nervous system, ChR2 can evoke spike trains with temporal precision (Boyden et al., 2005). Non-invasive and highly specific spatiotemporal manipulations of neural activity by ChR2 have been applied to elicit presynaptic neuron firing for functional mapping, recapitulate spike patterns, and distinguish spikes of a defined population from others (Kvitsiani et al., 2013; Lim et al., 2013; Pi et al., 2013). ChETA has also been used to evoke spikes with higher frequency (Gunaydin et al., 2010). Two major light-evoked activity silencers have been used to test the necessity of cellular activity in generating normal cortical activity and behavioral responses. First, eNpHR is an enhanced version of halorhodopsin, which is a chloride pump activated by yellow light (Gradinaru et al., 2008). Second, Arch is a proton pump activated by green light, which can drive large inhibitory currents (Idnurm and Howlett, 2001; Chow et al., 2010).

Another approach to enhance and reduce neuronal activity is pharmacogenetic tools such as designer receptors exclusively activated by designer drugs (DREADDs). DREADDs are genetically engineered muscarinic acetylcholine receptors, which are insensitive to endogenous acetylcholine but sensitive to the synthetic ligand, clozapine-*N*-oxide (CNO; Armbruster et al., 2007). DREADDs can be reversible and bidirectional. Upon administration of CNO, G_q_-coupled human M3 DREADD activates neurons likely through closure of KCNQ channels while G_i_-coupled human M4 DREADD inhibits neuronal activity presumably through GIRK channels (Armbruster et al., 2007). Advantages of DREADDs are that the effect is easily induced within 1 h after intraperitoneal injection of CNO and lasts for about 10 h (Wulff and Arenkiel, 2012). These features place DREADDs in a unique position over optogenetic tools due to the following reasons: (1) DREADDs have no requirement of laborious animal surgery while *in vivo* optogenetic approaches need to make a cranial window for photostimulation. (2) Chronic stimulation or inactivation can be easily achieved in DREADD systems by drug application while chronic photostimulation of optogenetic tools in live animals may not be practical due to necessity of long-lasting anesthesia. (3) Neurons in deep brain tissues can be manipulated in DREADD approaches while it is not trivial to deliver light to deep brain areas.

### CELL TYPE SPECIFIC GENOMICS

One of main challenges in neuroscience is being able to reveal gene expression profiles that underlie the assembly, organization, and function of a defined neuronal population. Genetic materials can be purified from fluorescently labeled target cells collected through physical methods such as laser-capture microdissection, fluorescence-activated cell sorting, and manual sorting. However, since these procedures cause physical damage and stress, the physiological condition and normal gene expression of cells can be disrupted when isolating cells of interest. Recent advances in genetic tagging methods overcome this obstacle and provide a way to obtain “intact” gene expression profiles in a select neuronal population. The translating ribosome affinity purification (TRAP) method uses EGFP-tagged ribosomal protein L10a to pull down mRNAs in a polysomal fraction with anti-GFP antibodies (Heiman et al., 2008). The RiboTag method is based on a similar idea, which utilizes HA (hemagglutinin)-tagged RPL22 (ribosomal protein L22) to purify translating mRNA with anti-HA antibodies (Sanz et al., 2009). Furthermore, the microRNA (miRNA) tagging and affinity purification (miRAP) method has been developed, in which the Argonaute 2 protein, a core component of the RNA-induced silencing complex that directly bind to miRNAs, is tagged with MYC peptides and the miRNAs are purified from the tissue homogenate using anti-MYC antibodies (He et al., 2012). If appropriate binding proteins are available, the genetic tagging strategy can be expanded to capture a subset of mRNAs localized in different cellular compartments or DNA fragments that have specific conditions and modifications.

### OTHERS

Other useful genetically encoded biosensors include ones that report synaptic events such as synaptic vesicle fusion and intracellular signaling in dendritic spines. The superecliptic pHluorin (SEP) is a pH-sensitive GFP whose fluorescence is quenched by the acidic condition within synaptic vesicles or endosomes and increases as the pH goes up (Miesenbock et al., 1998). The synaptopHluorins are SEP-based reporters, in which the SEP is fused with a synaptic vesicle protein such as synaptophysin (Granseth et al., 2006) or vesicular glutamate transport protein 1 (VGlut1; Balaji and Ryan, 2007), to study synaptic vesicle exocytosis and endocytosis. The SEP-tagged membrane bound receptors can also be used to analyze their membrane trafficking at synapses. The use of the SEP-tagged AMPA receptors (AMPARs) revealed that insertion of AMPARs into the plasma membrane of spines through endosomal exocytosis is an important step in long-term potentiation (Makino and Malinow, 2009). Several FRET-based intracellular signaling sensors have been employed to study signaling events in dendritic spines. These include a FRET-based CaMKII sensor and FRET sensors for small GTPase proteins such as Ras, Rho, and Cdc42 (Murakoshi and Yasuda, 2012).

## GENETIC TARGETING OF CORTICAL GABAergic INTERNEURON SUBTYPES

The most remarkable feature of GABAergic interneurons is the diversity in cell types, which is a basis of various inhibitory regulations in neuronal circuits (Markram et al., 2004; Klausberger and Somogyi, 2008). Therefore, cell type specific studies on development, anatomy, and function of GABAergic circuits are essential to understanding neuronal information processing regulated by cortical inhibition. However, it has been very difficult to tackle these questions due to limitations of strategies that can identify and manipulate cell types with precision and reproducibility.

GABAergic interneurons have been classified by several definitions such as axonal and dendritic morphology, connectivity, electrophysiological characteristics, gene expression pattern, developmental origins and features, and physiological function at a circuit level (Markram et al., 2004). These criteria have been used in combination to define more distinct cell types but there has been a long-standing debate regarding how a certain cell type is defined. In the context of neural circuits, the most reasonable definition for a cell type is probably the functional aspect since cells with the same function most likely share features defined by several other criteria. However, this is the most difficult definition to be used for classification of cortical interneurons because little is known about their function. Alternatively, it makes a lot sense to use gene expression profiles as a definition of a cell type because the expression of different kinds of genes should reflect distinct cellular phenotypes. However, at this point, this is not a realistic approach to categorize cortical GABAergic interneurons since comprehensive gene expression studies are far from complete. Nevertheless, in some cases, the use of several marker genes expressed in broad subpopulations of cortical GABAergic interneurons has successfully captured neuronal subtypes that display a consistent phenotype in distinct definitions; for instance, cortical interneurons expressing PV show a high correlation between physiologically defined fast-spiking phenotype and soma/proximal dendrite targeting (Kawaguchi and Kubota, 1997). The phenotypes of mature cortical interneurons, which serve as the criteria to identify cell types, can be accounted for by consequences of unique consecutive developmental events. It is possible that immature neuroblasts are progressively specified during development such that their potential to become a certain cell type is restricted over time. Therefore, unique developmental mechanisms that control cellular diversification, migration, and circuit integration may provide another definition to differentiate discrete cell types even before they become mature, although the link between the developmental history and the final cellular phenotype currently remains elusive.

Genetic targeting is probably the best approach to precisely and reliably identify and manipulate specific cell types. Along with the recent development of genetically encoded molecules such as FRET probes and optogenetic tools, genetic targeting methods have become more efficient and accurate, which dramatically promotes the investigation of the mysteries of neural circuits. Here, I will summarize several genetic methods that can be used to target select neuronal populations and introduce a project to systematically generate Cre driver lines that target subtypes and progenitors of cortical GABAergic interneurons.

### GENETIC TARGETING STRATEGIES

**FIGURE 3:**
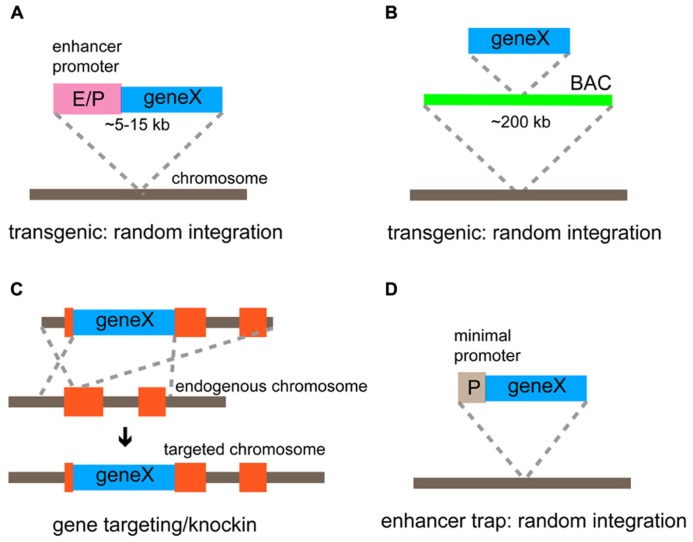
**Genetic strategies for targeted gene expression.** **(A)** Conventional transgenic strategy using a relatively small transgenic construct (5–15 kb) to express a gene of interest under the control of the defined enhancer/promoter. **(B)** BAC transgenic method using an engineered BAC construct (~200 kb) to express a gene of interest. *Cis*-regulatory elements (enhancers and a promotor) within BAC regulate the basic expression pattern of the gene. **(C)** Gene targeting/knockin approach that is achieved by the homologous recombination. A gene of interest is inserted at the endogenous locus and thus expressed under the control of endogenous gene regulatory elements. **(D)** Enhancer trap method using a construct comprising a minimal promoter and a gene of interest. The gene is expressed under the control of enhancers that influence its integration site.

There are two major strategies to express an exogenous gene in select neuronal types in the mouse cortex; the transgenic approach and the gene targeting/knockin approach. In the transgenic approach, a relatively small transgenic construct (~5–15 kb) containing a gene of interest and minimal *cis*-regulatory elements (enhancers and promoters; **Figure 3A**), or the recombinant bacterial artificial chromosome (BAC; ~200 kb) containing an exogenous gene and a nearly complete set of *cis*-regulatory elements (Heintz, 2001; **Figure 3B**) is randomly integrated into the host genome. The advantage of this strategy is that the process from vector construction to mouse generation is relatively straightforward and higher expression of an exogenous gene is expected as multiple copies of transgenes tend to be tandemly integrated into the genome. On the other hand, the transgenic method includes inherent disadvantages; the expression of a transgene may not completely recapitulate the expression pattern of the endogenous gene and may vary among transgenic mouse lines, which demands countless hours of screening to find the appropriate lines. Such incompleteness can be explained based on mechanisms for mammalian gene expression. First, coordinated activity of multiple *cis*-regulatory elements, which are distributed in the genome occasionally far away from the transcription start site, are essential to drive proper gene expression (Kapranov et al., 2007). Transgenic methods cannot guarantee that the full sets of regulatory elements are included. Therefore, even BAC constructs may not perfectly recapitulate the endogenous gene expression pattern. Second, unrelated enhancers and repressors surrounding the insertion site can impact the transcription of transgenes, resulting in ectopic or suppressed expression. Third, epigenetic modifications may silence or change the normal transcription of transgenes inserted into a foreign chromatin region. Although this drawback in the transgenic approach has annoyed researchers, who expect faithful recapitulation of the endogenous gene expression, the incomplete and partial expression of transgenes has been useful in some cases where a subset of cells within a certain population need to be examined. For example, transgenic mouse lines containing a GFP gene under the control of a glutamic acid decarboxylase (GAD) promoter show restricted expression in different subsets of inhibitory interneurons rather than ubiquitous expression in all GABAergic interneurons (Chattopadhyaya et al., 2004). The comprehensive generation of mouse lines expressing different types of genes such as reporters, sensors, and optogenetic tools, under the influence of Thy-1 promotor, is probably the best application of the random integration effect (Feng et al., 2000; Berglund et al., 2006; Zhao et al., 2008a; Chen et al., 2012; Ting and Feng, 2013). Although endogenous Thy-1 transcripts are highly expressed in many projection neurons, transgenes are expressed at a high level in a subpopulation of these neurons, ranging from 0.1% to almost all (Feng et al., 2000). These lines have made a tremendous contribution to studies of development and function of neural circuits; however, it is unclear to what extent a Thy-1 promoter is active in GABAergic interneurons. The transgenic approach can be feasible to generate mouse lines but extensive and careful screening is required to identify those that meet specific purposes.

Gene targeting/knockin utilizes homologous recombination in embryonic stem cells to introduce an exogenous gene into a specific genomic locus (Capecchi, 1989; **Figure 3C**). Unlike transgenic methods, gene targeting is the most precise and reliable strategy used to express transgenes faithfully following the expression pattern of the target gene because an exogenous gene of interest is inserted at an endogenous genomic locus with nearly intact gene regulatory environment. Recent advances in the fidelity of PCR enzyme, BAC recombineering technology (Warming et al., 2005), and embryological techniques using tetraploid blastocysts (Li et al., 2005) have facilitated the efficiency needed to generate knockin lines. Thus, the knockin approach is particularly useful if experiments need to deal with as many neurons within a certain population defined by the gene expression as possible with the greatest accuracy, and now not as laborious as before. However, there are disadvantages associated with the knockin strategy: (1) The expression level of a transgene can be low because of low copy number (maximally two copies in homozygous mice); (2) the expression of an endogenous gene in the target locus is either knocked out or down-regulated when a transgene is inserted at the translation start site or after the coding region of the targeted gene through an internal ribosomal entry site (IRES) or a 2A sequence to enable the bicistronic expression, respectively (Taniguchi et al., 2011).

An additional genetic strategy to target cell types is the enhancer trap, in which an enhancer trap cassette containing a minimal promoter and a transgene is randomly incorporated into the genome (**Figure 3D**). The transgene shows a distinctive expression pattern depending on the insertion site that is affected by a unique set of enhancers. The most successful example using this strategy is the GAL4 (the yeast TF)/UAS (GAL4-binding upstream activation sequence) system in fruit flies (Fischer et al., 1988; Brand and Perrimon, 1993), where a library of GAL4 enhancer trap lines is systematically and thoroughly screened. The enhancer trap approach has also been applied to mice, though not yet popular, to introduce different genes such as GAL4, LacZ, and Cre into various subsets of neurons (Kelsch et al., 2012). Notably, enhancer trap vectors have recently been delivered through lentiviruses to enhance genomic integration of a single copy (Kelsch et al., 2012). The major difficulty in this approach is that systematic characterization of a large number of enhancer trap lines is required to find truly useful ones.

### BINARY EXPRESSION SYSTEMS

The final goal of genetic targeting is to express reporters and effectors in a defined neuronal population to study the development and function of neural circuits. Genetically encoded molecular tools can be expressed directly from transgenic constructs or an endogenous gene locus targeted by a knockin strategy. However, this strategy does not always produce a high enough level of transgene expression, which can make these mouse lines totally useless. To overcome this issue, several binary expression strategies, in which the expression of genetic tools for neural circuit studies is regulated by two transgenes provided by breeding of driver lines and responder lines, have been developed. The combinatorial power of the binary system is absolutely essential for systematically studying different aspects of neural circuits. Currently, there are two major binary expression systems, transactivation-based systems and recombination-based systems.

**FIGURE 4:**
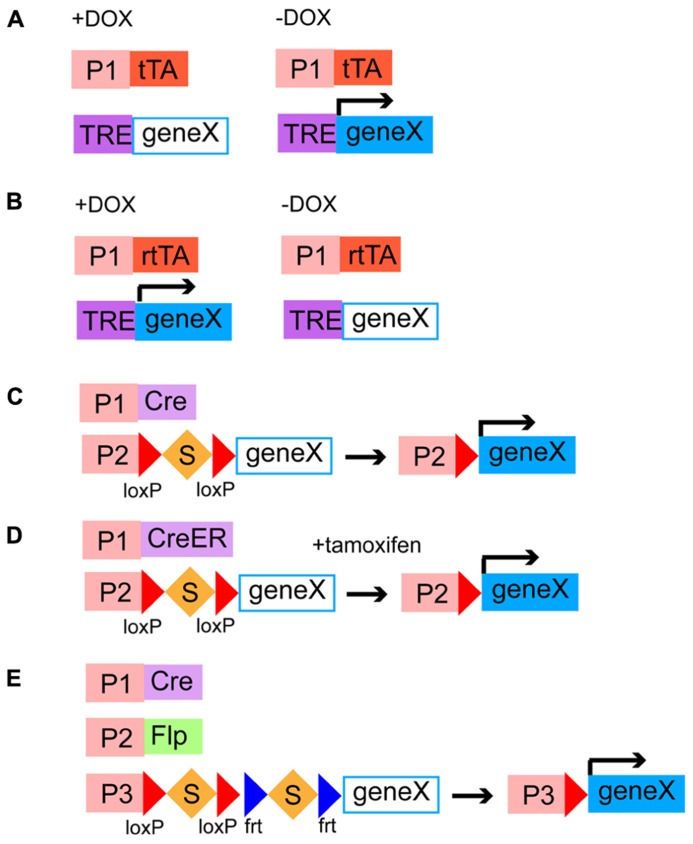
**Binary gene expression systems.** **(A,B)** Transactivation-based systems of tetracycline-regulated transactivators and a TRE promoter. tTA or rtTA is expressed in specific cell populations by different genetic targeting strategies. tTA and rtTA drive the expression of a gene of interest downstream to a TRE promoter in the absence **(A)** and presence **(B)** of DOX, respectively. P1, cell type specific promoter; DOX, doxycycline, a tetracycline analog; TRE, a tetracycline-responsive element. **(C–E)** Site-specific DNA recombinase-based systems. **(C,D)** Cre or CreER is expressed in specific cell types by distinct genetic targeting strategies. Cre removes the transcriptional stop cassette and then a gene of interest is expressed under the control of a ubiquitous promoter **(C)**. CreER is activated by addition of tamoxien and then excises the stop cassette, leading to the expression of a gene of interest **(D)**. S, a transcriptional stop cassette; P1, a cell type specific promoter; P2, a ubiquitous promoter. **(E)** Intersectional strategy using a Cre/Flp-dependent dual responder line. Cre and Flp are expressed in distinct neuronal classes, which have partially overlapping population. The intersectional population that expresses both Cre and Flp can drive the expression of a gene of interest under the control of a ubiquitous promoter. P1 and P2, cell type specific promoters; P3, a ubiquitous promoter.

In transactivation-based systems, distinct driver lines express transcriptional activators in different patterns and responder lines include genes of interest preceded by promoter sequences that bind activators. The advantage of this system is that transcriptional amplification can increase the level of the transgene expression. One of the most common transactivation-based systems is the GAL4/UAS system, which has been frequently used in fruit flies but not in mice (Fischer et al., 1988; Brand and Perrimon, 1993). In mice, the most popular binary transactivation system is the tetracycline-inducible transgene expression. The bacterial tetracycline-regulated transactivator (tTA), which is supplied by driver lines, drives the expression of exogenous genes under the control of the tetracycline-responsive element (TRE) promoters, which originate from responder lines, when tetracycline is not present (Berens and Hillen, 2004; **Figure 4A**). The tTA has been modified to generate the rtTA, which is active only in the presence of tetracycline (Berens and Hillen, 2004; **Figure 4B**). Thus, in this binary expression system, the level, density, and timing of the transgene expression can be controlled, choosing the amount and administration timing of tetracycline. In general, systems relying on transactivation are reversible, which may be advantageous for some purposes.

Site-specific DNA recombination-based systems require driver lines expressing a DNA recombinase in different neuronal populations under the control of *cis*-regulatory elements and responder lines containing a ubiquitous strong promoter and a gene of interest separated by a transcription stop cassette flanked by two recombinase recognition sites (Dymecki and Kim, 2007). In responder lines, transgenes are usually inserted into well-defined ubiquitous gene loci such as ROSA26 and H11 (Madisen et al., 2010; Tasic et al., 2011) so that they are universally expressed in all types of neurons. When a DNA recombinase and a recombinase-dependent transgene cassette coexist in the same cells, the STOP sequence is deleted by the site-specific homologous recombination, leading to the irreversible transgene expression (Madisen et al., 2010; Taniguchi et al., 2011). The Cre/loxP system (**Figure 4C**) and the Flp/frt system are two major representatives of binary recombination-based systems in mice (Dymecki, 1996; Dymecki and Kim, 2007). Other types of yeast or phage recombinases such as Dre, PhiC31, B3, and KD have been further applied to the binary gene expression system in mice although these need to be validated for efficiency and toxicity (Anastassiadis et al., 2009; Nern et al., 2011). Combination of multiple recombination systems will allow us to restrict the expression of transgenes in more selective populations (intersectional methods, see below) and combinatorially express different genetically encoded molecular tools in the same cells. To endow these systems with temporal control beyond cell type specificity, Cre and Flp have been engineered to fuse with a modified estrogen-binding domain of the estrogen receptor, generating CreER (**Figure 4D**) and FlpER (Feil et al., 1997; Hunter et al., 2005). CreER and FlpER are located in the cytoplasm by default but translocated into the nucleus to induce recombination only in the presence of tamoxifen, a synthetic estrogen analog, thus being able to limit the recombination activity in a certain time window (Feil et al., 1997; Hunter et al., 2005; **Figure 4D**). This has been particularly useful in studying whole developmental events of certain temporal cohorts of neurons. Besides temporal control, inducible Cre and Flp can be activated only in a small number of cells within a given population, with lower amount of tamoxifen, which enables experiments at a single cell level (Taniguchi et al., 2011). Some drawbacks associated with the use of inducible recombinases are that high recombination efficiency is compromised, administration of tamoxifen can be toxic to embryos and pregnant females and cause behavioral abnormality. Because of versatility and reliability of the Cre/loxP system, there is no doubt that systematic generation of Cre/CreER driver lines will be instrumental in elucidating complex development and function of neural circuits (Dymecki and Kim, 2007; Taniguchi et al., 2011). The NIH Blueprint for Neuroscience Research^1^ and GENSAT^2^ has supported systematic efforts in making a collection of Cre driver lines (Gong et al., 2007; Taniguchi et al., 2011). The efficiency and expression level of responder lines are also critical factors to make the Cre/loxP system useful. A project spearheaded by the Allen Institute has recently developed a series of responder lines containing markers, sensors, and transducers that meet these requirements (Madisen et al., 2010, 2012). A major trick used in their mouse lines is the addition of a woodchuck hepatitis virus posttranscriptional regulatory element (WPRE) sequence, which facilitates mRNA transportations from the nuclei to the cytoplasm and mRNA stability, to the 3′ untranslated region (Madisen et al., 2010). The alternative to responder lines for the expression of genes of interest is to use Cre- or Flp-dependent viruses. Recombinant adeno-associated viruses and lentiviruses have been made Cre or Flp-dependent and successfully combined with driver lines expressing DNA recombinases (Tiscornia et al., 2004; Atasoy et al., 2008; Kuhlman and Huang, 2008). Recombinase-dependent viral strategies combined with Cre or Flp drivers confer spatiotemporal control on the transgene expression without use of inducible ones by selecting injection sites and timing.

More specific cell types can be defined by expression of two or more genes. For instance, at least some Martinotti cells are delineated by expression of SOM and CR (Fogarty et al., 2007; Sousa et al., 2009). A select subtype with specific laminar distribution can be characterized by both the expression of a marker gene for an interneuron subtype and its birth date, since there is a strong correlation between the birth timing and the laminar position (Miyoshi et al., 2007). In this case, genes expressed in progenitor cells or intermediate progenitor cells are useful to tag a certain time window of the birth date. To target more minute neuronal populations, an intersectional method using different Cre and Flp lines is powerful and useful (Kim and Dymecki, 2009; Taniguchi et al., 2011; **Figure 4E**). This method requires three genetic components; (1) Cre/CreER driver lines expressing Cre/CreER under the control of *cis*-regulately elements of gene A, (2) Flp driver lines that express Flp following the expression pattern of the endogenous gene B, and (3) dual responder lines containing a gene of interest and dual STOP cassettes with loxP and frt sites. When these three alleles are brought together, by breeding animals, the exogenous gene of interest is turned on only in cells that have expressed Cre/CreER and Flp sequentially or simultaneously, where dual STOP cassettes are removed. Therefore, the intersectional approach is promising for labeling and manipulation of more specific and narrower populations defined by combinations of neurochemical and developmental features.

### GENERATION OF CRE/CREER DRIVER LINES TARGETING GABAERGIC INTERNEURON SUBTYPES

**FIGURE 5:**
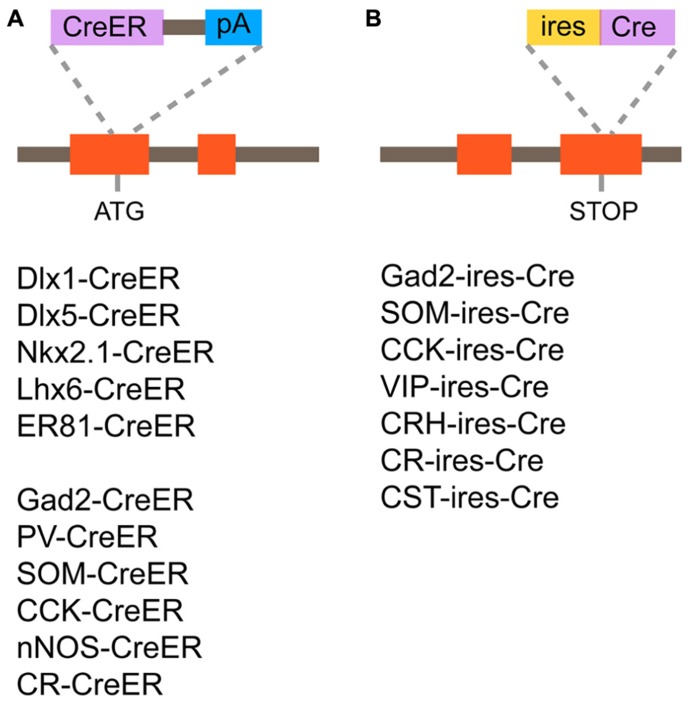
**Cre/CreER knockin driver lines targeting GABAergic mature interneurons and progenitors.** **(A)** Design of CreER knockin strategy and list of CreER driver lines. CreER is inserted at the translation initiation codon of the target gene locus. **(B)** Design of Cre knockin strategy and list of Cre driver lines. Cre is inserted immediately after the translation STOP codon followed by the internal ribosomal entry site (ires) sequence.

Recently, systematic efforts supported by the NIH Blueprint for Neuroscience Research have been made to generate and characterize nearly 20 Cre or CreER knockin driver lines targeting GABAergic mature neurons and embryonic progenitors (Taniguchi et al., 2011). In this project, two categories of genes have been targeted; (1) TFs, which are expressed in progenitors of the MGE during embryogenesis (Dlx1, Dlx5, Lhx6, Nkx2.1, and ER81; **Figure 5A**); (2) terminal differentiation markers expressed in all (gad2) or broad subtypes of mature and/or developing GABAergic interneurons (PV, SOM, VIP, CR, CCK, corticotropin releasing hormone, cortstatin, and nNOS; **Figures 5A,B**). In the TF lines, CreER was inserted at the translation initiation codon of the target gene locus to achieve the maximal expression level (**Figure 5A**). In the terminal differentiation marker lines, Cre was integrated immediately after the translation STOP codon followed by the IRES sequence as well as at the start codon of the target endogenous gene (**Figure 5B**). The extensive characterization of these knockin lines by crossing with Cre-dependent GFP and RFP reporters have demonstrated that in almost all cases recombination patterns faithfully recapitulate those of endogenous gene expression. For instance, in gad2-ires-Cre lines, more than 90% of cells expressing GFP induced by Cre activity are Gad67 (a pan inhibitory interneuron marker) positive and the fraction of Gad67 positive cells expressing GFP is more than 90%, indicating a high degree of specificity and efficiency. Inducible Cre lines also show high specificity and reasonable efficiency. In gad2–CreER lines, the density of reporter expression can be adjusted by tamoxifen dosage and almost all major interneuron subtypes are captured, as indicated by coexpression with PV, SOM, CR, VIP, and nNOS. It is also shown that other Cre drivers successfully target virtually all non-overlapping broad subtypes of cortical interneurons, such as SOM, VIP, and CCK, with great precision and efficiency.

In SOM-ires-Cre drivers, major dendrite-targeting interneuron subclasses are captured in the cortex and hippocampus. In the cortex, the dense axonal band of Martinotti cells that target apical tufts of pyramidal cell dendrites is visualized in L1. In the hippocampus, the prominent axonal band of O-LM cells that form synaptic connections with apical tufts of CA1 pyramidal neurons is labeled in the stratum lacunosum moleculare. SOM starts to be expressed in developing interneurons, which just exit the SVZ of the MGE, from mid-gestation onward (Batista-Brito et al., 2008). Consistent with this fact, tangentially migrating neurons toward the cortex are labeled in the MZ of the basal forebrain in SOM-ires-Cre mice. These labeled neurons are probably developing immature SOM neurons rather than neurons that transiently express SOM during development because in the mature cortex most neurons with reporter expression are still SOM positive. Thus, SOM-ires-Cre driver lines provide reliable genetic access to developing and mature SOM neurons to study their migration, assembly into neural circuits and functions.

VIP-ires-Cre driver lines specifically and efficiently capture VIP-expressing neurons that are derived from the CGE. In these animals, typical VIP neurons that extend vertically oriented dendrites and axons are visualized. Unlike SOM neurons, VIP neurons show no axonal arborization in L1 in the cortex. In the hippocampus, VIP neurons display two distinct axonal bands in stratum pyramidale and stratum oriens. Electrophysiological studies indicated that neurons labeled in this line show unique intrinsic properties similar to those observed in rat VIP neurons, confirming specificity from the physiological side. VIP expression can be seen from the neonatal stage and developing VIP neurons are also labeled in the immature cortex of VIP-ires-Cre drivers. Thus, VIP-ires-Cre lines are useful for studying development and function of CGE-derived interneurons.

CCK-expressing neurons comprise one class of basket interneurons that innervate the soma and proximal dendrites of pyramidal neurons. When CCK-ires-Cre drivers are simply combined with Cre-dependent reporters, no selective labeling of CCK-expressing neurons occurs, perhaps because CCK is not only expressed in GABAergic interneurons but also pyramidal neurons. To overcome this limitation, the intersectional method is applied to target GABAergic CCK-expressing neurons in the cortex. Dlx5/6-Flp is a transgenic line expressing the Flp in most cortical GABAergic interneurons. Combination of CCK-ires-Cre, Dlx5/6-Flp and FSF-LSL-GFP can specifically visualize GABAergic CCK-expressing neurons. In the cortex, CCK and PV (the other class of basket interneurons) terminals are differentially labeled around the same pyramidal neuron. Therefore, the intersectional method provides genetic access to GABAergic CCK-expressing neurons, facilitating studies of their migration, circuit integration, and function.

Genetic fate mapping using TF lines that express CreER in MGE progenitors has been used to study how lineage and birth timing contribute to specification of interneuron subtypes and the subsequent migration, circuit integration and functional emergence of postmitotic interneurons (Joyner and Zervas, 2006). The Nkx2.1-CreER line targets SVZ and VZ progenitors in the MGE and POA but not the LGE and CGE. This driver line is useful to globally visualize and manipulate MGE and POA progenitors and their derivatives at different time points. In contrast, ER81 is expressed in the most ventral portion of the MGE and the most dorsal part of the POA (Flames et al., 2007). Thus, The ER81-CreER driver will provide a genetic experimental system to examine what types of interneurons are produced from subdivisions of the MGE and POA, which will elucidate the spatial organization of different types of interneuron progenitors.

This first round effort to generate Cre driver lines targeting GABAergic interneurons has made it possible to dissect diverse and complex cortical GABAergic circuits at a much finer level and has changed the experimental approach used to study the GABAergic system. It has been proven that in combination with recent innovation of genetically encoded molecular tools, these lines are extremely instrumental in studying connectivity, circuit and system level function, and origin and development, of cortical interneuron subtypes (Miyamichi et al., 2011; Adesnik et al., 2012; Gentet et al., 2012; Kvitsiani et al., 2013; Taniguchi et al., 2013). Future efforts will be to generate more cell type specific Cre and Flp lines to capture more select populations. Since GABAergic inhibition globally plays a critical role throughout the nervous system, these drivers will also be useful for dissecting many other brain circuits outside the cortex.

## ORIGIN, ORGANIZATION, AND FUNCTION OF CHANDELIER CELL

One of the most striking features in the GABAergic system is that distinct subtypes form synaptic connections onto different subcellular compartments of excitatory pyramidal neurons (Somogyi et al., 1998; Buzsaki et al., 2004). Among various subtypes of cortical interneurons ChCs show the most unique subcellular synapse specificity, distinguishing them from others. These cells exhibit a characteristic axonal arbor with short vertical rows of presynaptic boutons (cartridges), which resembles the candles of an old-fashioned chandelier, and these cartridges exclusively innervate AISs of excitatory pyramidal neurons (Szentágothai and Arbib, 1974; Somogyi, 1977). Since AISs are critical sites that generate action potentials, it has been proposed that ChCs exert the most powerful influence on cortical circuit activity. ChCs are rare, consisting of a small fraction of all GABAergic interneurons in the cortex. Because of this minority, even Santiago Ramon y Cajal, the father of neuroanatomy, missed ChCs and never described their striking synaptic cartridges. It was almost four decades ago that Szentagothai first discovered these cells and named them ChCs based on their peculiar morphology (Szentágothai and Arbib, 1974). His speculation was that synaptic cartridges are formed along apical dendrites of pyramidal neurons, but later that turned out to be wrong. Somogyi subsequently demonstrated that neurons, which show morphological characteristics similar to ChCs, are the source of presynaptic boutons formed on AISs of pyramidal neurons, and called them axo-axonic cells (AACs; Somogyi, 1977). Thus, both ChCs and AACs have been interchangeably used as a terminology in the field of cortical GABAergic circuits. ChCs have been found not only in the neocortex but also in the allocortex and the basolateral amygdala (Somogyi, 1977; Kosaka, 1983; Soriano and Frotscher, 1989; Schmidt et al., 1993). Although ChCs are widely distributed in the pallial telencephalon, the distribution of complex synaptic cartridges detected by immunostaining against GABA transporter 1 (GAT-1) is shown to differ in distinct cortical regions and layers (Inda et al., 2009). These studies indicated that denser complex cartridges are in the piriform cortex and entorhinal cortex than in the neocortex (Inda et al., 2009). However, it is unclear whether such differential distribution of complex cartridges stems from the difference in the number of ChCs or cartridges derived from a single ChC. An anatomical and structural analysis of an axonal arbor of a single ChC provides an insight into how ChC outputs impact cortical network activity. This has been traditionally done with Golgi method, intracellular dye filling, and EM reconstruction, all of which are time-consuming techniques and thus can only analyze a limited number of cells (Somogyi et al., 1982; Li et al., 1992; Lund and Lewis, 1993; Martinez et al., 1996; Krimer and Goldman-Rakic, 2001; Inda et al., 2009). Recent advances in genetic methods have accelerated identification and labeling of ChCs and allowed a detailed analysis of anatomical connectivity of an axonal arbor from a single ChC. The mosaic analysis with double markers (MADM) technique enables labeling of a single L2 ChC in the somatosensory cortex and an efficient reconstruction of individual axonal arbors and AISs by immunostaining for GFP and AIS markers (Inan et al., 2013). This study concluded that a single ChC has approximately a hundred cartridges, which innervate 35–50% of pyramidal neurons within an area covered by an axonal arbor. It was also shown that each cartridge of a ChC contains 3–5 boutons on an AIS, and on average four ChCs innervate one pyramidal neuron. Dense and overlapping innervation of pyramidal neurons by ChCs may exert a widespread and effective influence on local circuit activity.

Because of their GAD immunoreactivity, ChCs have traditionally been considered inhibitory interneurons (Somogyi et al., 1983). Consistent with this view, occasional *in vivo* recordings provided results implying an inhibitory role of ChCs in control of neural circuit activity. It has been shown that hippocampal ChCs spike in antiphase to pyramidal neurons during theta wave oscillation, and fire right before pyramidal neuron spiking during sharp wave associated ripples (Klausberger et al., 2003). Whole-cell recordings from ChCs in the rat somatosensory cortex showed that ChCs are robustly recruited into the cortical circuits when the overall network activity becomes high, although they rarely fire in a quiescent state (Zhu et al., 2004). This suggests that ChCs may operate to suppress excessive excitation via their powerful inhibitory synapses on AISs of pyramidal neurons. However, unexpectedly, recent studies using different recording techniques including the gramicidin perforated patch recording and the cell-attached recording demonstrated that ChCs have a depolarizing effect on postsynaptic pyramidal neurons at resting membrane potential in mouse, rat, and human neocortical slice preparations (Szabadics et al., 2006; Molnar et al., 2008; Woodruff et al., 2011). Furthermore, it was also observed that ChC-triggered postsynaptic depolarization causes disynaptic suprathreshold excitation in neighboring neurons, suggesting that a single ChC can directly drive neuronal spikes at the AISs of multiple pyramidal neurons (Szabadics et al., 2006; Molnar et al., 2008; Taniguchi et al., 2013). This depolarizing and potentially excitatory effect of ChCs has been explained by the depolarized axonal GABA_A_ reversal potential, which is generated and maintained through the high expression of the chloride-importing cotransporter NKCC1 in addition to reduced expression of the chloride-extruding cotransporter KCC2 (Szabadics et al., 2006; Khirug et al., 2008). Although many lines of evidence have supported the idea that cortical ChCs may exert excitatory influences on their target pyramidal neurons, a recent study using the non-invasive field potential recordings indicated that hippocampal ChCs are predominantly hyperpolarizing (Glickfeld et al., 2009). The opposite results perhaps stem from different brain regions where ChCs are recorded, and different recording techniques. To get a definitive answer, future studies will require careful comparisons of ChCs from different brain regions, ideally using non-invasive approaches *in vivo*. The use of genetically encoded optical sensors and effectors may provide a novel and ideal approach to address this issue.

**FIGURE 6:**
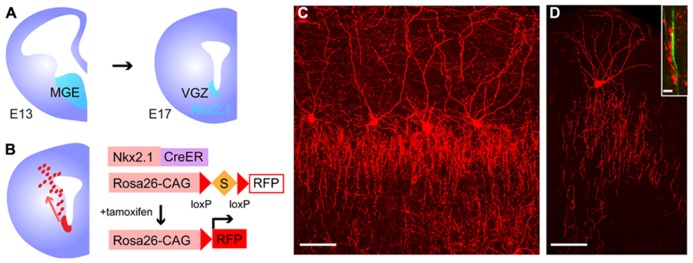
**Genetic targeting of chandelier cells.** **(A)** Expression of Nkx2.1 in the ventral forebrain during mid-late gestation. Nkx2.1 is expressed in the MGE during mid-gestation. Even after the MGE became morphologically invisible, Nkx2.1 continues to be expressed in the VGZ during late gestation. **(B)** Genetic fate mapping of ChC progenitors using Nkx2.1-CreER driver lines and Cre-dependent reporters. CreER-mediated excision of a stop cassette is induced by addition of tamoxifen and results in RFP expression in Nkx2.1-expressing progenitors in the VGZ. Red dots in the left scheme show migrating neurons from the VGZ. **(C)** A cluster of L2 ChCs in the adult medial prefrontal cortex captured by genetic fate mapping strategy. **(D)** A single L2 ChC in the morter cortex. Inset shows a synaptic cartridge (in red) innervating an axon initial segment (in green) stained with an anti-phospho IκB antibody. Scale bars: 50 μm in **(C,D)** 5 μm in inset of **(D)**.

Although having attracted broad interest from many neuroscientists because of striking morphology and possible significance in cortical circuits, little has been known about origin, development, and anatomical details such as cellular distribution and input/output connectivity, of ChCs. Because of the inability to manipulate a population of ChCs, comprehensive understanding of this cell type has been hampered. However, the recent genetic fate mapping of Nkx2.1-expressing progenitors in the late embryonic subpallium using Nkx2.1-CreER mice has demonstrated that ChCs mainly derive from the ventral germinal zone of the lateral ventricle (VGZ), an Nkx2.1-expressing remnant of the MGE, during late gestation (Taniguchi et al., 2013; **Figures 6A,B**). Reproducible labeling of spatially and temporally defined ChC progenitors in the VGZ enabled to not only examine whole developmental events but also to study laminar and area distribution of a group of ChCs in the adult cortex (**Figures 6C,D**). The results have shown that ChC neuroblasts migrate with specific routes and schedules, and settle in upper L2 and L5/6 of the cortex. These ChCs are significantly more enriched in the frontal cortex including the cingulate, prelimbic, and infralimbic cortices compared to the sensory cortex, such as the visual and auditory cortices. Immunohistochemical analysis demonstrated that only a subset of ChCs captured by this genetic fate mapping technique is immunoreactive for PV, which is contrary to the generally believed idea that ChCs are PV-expressing neurons. In addition to uncovering embryonic origin and fundamental organization throughout the cortex, of ChCs, importantly, this study established reliable genetic access to ChCs, which allows targeted introduction of genetically encoded tools in ChCs. Future experiments taking advantage of targeted manipulations in ChCs are expected to address the remaining critical questions, including the following issues. (1) Molecular mechanisms that determine ChC identity are totally unknown. Candidate genes need to be identified and functionally tested in ChC progenitors. (2) Cellular and molecular mechanisms that enable ChCs to establish characteristic axonal arbors and synaptic specificity at AISs are totally unknown. The first step will be to describe the whole process of axonal development at a single cell level. (3) Although the laser scanning photostimulation technique suggested that L2/3 ChCs receives excitatory inputs from L2/3 and 5a, and inhibitory inputs from L1 and L2/3 (Xu and Callaway, 2009), cell types that send inputs to ChCs need to be clarified. More precise and systematic input analysis can be done with monosynaptic retrograde tracing using pseudotyped RVs. (4) Functions of ChCs at circuit and behavioral levels remain unknown. Recording and manipulating ChC activity will be performed using optogenetic tools. (5) Defects in ChCs have been implicated in neurological diseases such as schizophrenia and epilepsy (DeFelipe, 1999; Lewis, 2010). Structural and functional analysis of ChCs in disease model mice will provide a deep insight into pathogenesis of brain disorders. These questions raised above can be applied to all subtypes of GABAergic interneurons. Since ChCs are the most distinctive and purest cell type among many GABAergic interneuron subtypes, they will serve as an excellent model to ask cell type specific questions from specification and development to function.

## CONCLUSION

The extreme complexity of cortical circuits has been a huge obstacle to gaining a precise understanding of how the brain is constructed and operates. It is obvious that the diversity of cortical GABAergic interneurons in their morphological, physiological, histochemical, and anatomical properties contributes to the structural and functional complexity of cortical networks. Thus, dissecting specification, development, connectivity, and function of each GABAergic interneuron subtype will be a key topic of investigation to understand cortical function. Simultaneous development of genetically encoded molecular tools and genetic targeting of GABAergic interneuron subtypes has been critical to address these questions. In particular, Cre/CreER driver mouse lines targeting embryonic progenitors and major subtypes of mature GABAergic interneurons have provided the most powerful and versatile strategy to interrogate cell type specific issues. Although we have just obtained genetic access to broad subtypes of GABAergic interneurons, more distinct and homogenous cell types such as NGFCs or Martinotti cells has never been captured by a current set of Cre/CreER drivers, except for ChCs targeted by use of Nkx2.1-CreER lines. Gene expression profiling of known subtypes, intersectional strategies using different Cre and Flp drivers, and analysis of temporal, spatial, and lineage mechanisms to diversify cortical interneurons will innovate additional methods to capture such smaller and purer populations. ChCs will become a pioneer model to study development and function of a pure cell type. Abnormal operation of GABAergic circuits has been implicated in many brain disorders such as epilepsy, schizophrenia, and autism. Systematic analysis of each cortical interneuron subtype in disease model mice will elucidate cellular and molecular pathogenesis, leading to innovation of novel therapeutic strategies. Together, genetic targeting of GABAergic interneuron subtypes will break down complex circuit organization into relatively simple and homogenous units and help to reveal the principle of cortical organization and function.

## Conflict of Interest Statement

The author declares that the research was conducted in the absence of any commercial or financial relationships that could be construed as a potential conflict of interest.

## Footnotes

1 http://neuroscienceblueprint.nih.gov/

2 http://www.gensat.org/cre.jsp
